# Corneal eccentricity and related factors in Chinese children and adolescents with astigmatism

**DOI:** 10.1186/s12886-025-04402-3

**Published:** 2025-10-07

**Authors:** Lixing Zhou, Jieli Mao, Min Cao, Chuanyan Wu, Mengyu Zhu, Stephen J. Vincent, Aiqin Xu, Ruzhi Deng

**Affiliations:** 1 National Clinical Research Center for Ocular Diseases, Eye Hospital, Wenzhou Medical University, Wenzhou, 325027 China; 2 Department of Ophthalmology, Quzhou People’s Hospital, Quzhou, 324000 China; 3 Centre for Vision and Eye Research, Queensland University of Technology, Brisbane, Queensland Australia

**Keywords:** Corneal eccentricity, Corneal astigmatism, Corneal topography

## Abstract

**Objective:**

To quantify the distribution of anterior corneal eccentricity (e value) and identify associated factors in Chinese children and adolescents with astigmatism.

**Methods:**

Corneal topography data obtained using the Medmont E300 were retrieved from 961 participants aged 3 to 18 years, with corneal astigmatism (ΔK) ≥ 2.00 D. Data analysis included anterior corneal e values along both flat and steep meridians at 1–10 mm chord lengths, as well as the device-reported mean e values for the flat and steep meridians (9.35 mm chord dimater); anterior corneal keratometry readings (flat/steep K); ΔK; and refractive error. Demographic factors, including age and sex, were also analyzed.

**Results:**

This study included 961 right eyes (mean age: 7.9 ± 2.9 years; 56% male). The mean e values were 0.73 ± 0.10 for the flat meridian and 0.51 ± 0.21 for the steep meridian. Along the flat meridian, e values decreased and stabilized with increasing chord length, while along the steep meridian, a U-shaped trend was observed. The mean flat e was correlated with the flat K (β = −0.013, *P* < 0.001) and ΔK values (β = 0.037, *P* < 0.001), whereas the mean steep e was only associated with sex (β = −0.043, *P* = 0.001) in multivariate analyses.

**Conclusion:**

In Chinese children and adolescents with moderate to high corneal astigmatism, the anterior cornea demonstrates aspheric characteristics with distinct meridional asymmetry. The flat e value may was associated with corneal curvature (flat K and ΔK values), whereas the steep e value was not associated with these parameters.

**Supplementary Information:**

The online version contains supplementary material available at 10.1186/s12886-025-04402-3.

## Introduction

The anterior corneal surface is aspheric and often modeled as a conic section. [1]. This shape is characterized by the apical radius and the eccentricity (e value) [2] which quantifies the change in corneal curvature from the center to the periphery [2]. Reported anterior corneal e values vary considerably across studies, partly due to methodological differences, especially the chord length used for calculation (e.g., 6.0 mm vs. 9.0 mm) [3, 4]. Although longer chord lengths generally yield higher e values [3, 4], this factor alone does not fully explain the observed variability. In addition, meridional differences in e values have been reported, suggesting meridional asymmetry in corneal shape between principal meridians, which may further contribute to inter-study variations [5, 6]. Regional asymmetry in corneal shape has also been observed in Chinese emmetropes and myopic astigmats aged 10 to 45 years [7], indicating that corneal asphericity varies across different corneal regions.

The anterior corneal e value has important clinical implications. A precise understanding of central and peripheral corneal shape is critical in rigid contact lens fitting—including corneal, reverse-geometry orthokeratology, and scleral lenses. Relying solely on central corneal curvature or axial power for rigid lens fitting can lead to poor alignment and adverse physiological outcomes. Notably, a recent study [8] demonstrated that the corneal e value was significantly associated with the treatment zone area in orthokeratology, underscoring its role in lens design. Additionally, e values can inform the diagnosis of keratoconus [9, 10] planning for corneal refractive surgery [11, 12] and calculation of the optimal intraocular lens power [13, 14].

Previous research on corneal e values has primarily focused on adults [4, 15–17]. However, corneal shape changes with age, typically showing decreasing e values over time [4, 18], highlighting the need for data specific to pediatric populations. Ethnic differences in corneal asphericity have also been reported [19]. Moreover, meridional variations in corneal shape [5, 6] which may impact contact lens fitting and visual performance, remain underexplored in younger populations. Astigmatism ≥ 2.00 D is often considered clinically significant [20, 21] and many individuals with high astigmatism require contact lenses for optimal vision correction [22, 23]. Despite the high prevalence of astigmatism among Chinese children and adolescents [24, 25], little is known about the meridional distribution of e values in this group, especially in those with high astigmatism. Although a few studies have reported anterior corneal e values in Chinese children, they were limited by small sample sizes [3] and the inclusion of only low levels of corneal astigmatism (< 1.50 D) [5]. Furthermore, while sex [18, 26] and refractive error [4, 15] have been linked to corneal morphology, their association with corneal e values remains unclear in younger, astigmatic populations.

To address these gaps, we investigated meridional anterior corneal e values in a large cohort of Chinese children and adolescents with moderate-to-high astigmatism (≥ 2.00 D). We assessed the association between corneal e values with the magnitude of astigmatism, meridional keratometry, age, sex, and refractive error—factors known to influence corneal shape and optical performance. Based on previous evidence, we hypothesized that a greater magnitude of astigmatism would be correlated with higher corneal e values and also explored whether e values varied with corneal meridian and other ocular parameters.

## Methods

This retrospective study was conducted at the Eye Hospital of Wenzhou Medical University. It adhered to the principles of the Declaration of Helsinki and was approved by the Ethics Committee of the Eye Hospital of Wenzhou Medical University (2022-119-K-90).

Data were obtained from 961 consecutive patients who underwent corneal topography examinations using the Medmont E300 (Medmont Pty Ltd, Victoria, Australia) between January 2019 and December 2023. The study population was selected based on the following inclusion criteria: (1) age range 3 to 18 years, (2) ΔK ≥ 2.00 D in the measured eye, and (3) topographic data acquisition ≥ 6 mm chord length coverage. Exclusion criteria were: (1) incomplete or suboptimal quality topographic images (as determined by the built-in quality assessment algorithm), (2) unavailable refractive data, (3) clinical diagnosis of keratoconus or other corneal pathologies, (4) history of ocular surgery or significant trauma, (5) regular soft contact lens wear within the preceding 3 months, and (6) any history of rigid gas-permeable contact lens use, including orthokeratology lenses.

For each participant, key topographic parameters, including anterior corneal e values along both flat and steep meridians at 1–10 mm chord lengths, anterior corneal keratometry readings (flat and steep K), ΔK, were directly extracted from the topography software.

To facilitate comparisons with previous research, the mean e values along both the flat and steep meridians were obtained directly from the Medmont E300 using its default settings (a chord length of 9.35 mm). This setting was chosen to maintain consistency with prior studies using the same device.

Corneal astigmatism was classified by the steep meridian axis as follows: with-the-rule (WTR) from 60° to 120°, against-the-rule (ATR) from 0° to 30° or 150° to 180°, and oblique from 31° to 59° or 121° to 149°, consistent with established clinical definitions [27].

Refractive error was measured using cycloplegic refraction when available, with spherical equivalent (SE) obtained by subjective refraction or retinoscopy depending on participant cooperation. If cycloplegic refraction was not performed, non-cycloplegic methods were used.

Only data from the right eye from each participant were analyzed. Statistical analysis was undertaken using SPSS software (version 26.0; IBM, Armonk, NY, USA). Given the large sample size (*n* = 961), formal normality testing was deemed unnecessary due to the central Limit theorem, supporting the use of parametric tests without this prerequisite. Corneal e values along the two principal meridians within the 1–10 mm chord length range were described using the mean ± standard deviation (SD), 95% confidence intervals, and various percentiles. Other continuous data were also expressed as the mean ± SD, while categorical data were presented as frequencies (percentage). The t-test was used for two-group comparisons, and analysis of variance (ANOVA) with post-hoc Tukey tests was applied for multiple-group comparisons. The Tukey post-hoc test was selected to control for type I error across multiple pairwise comparisons, providing a conservative adjustment for multiple testing. Simple and multiple linear regression analyses were conducted to assess the relationships between flat/steep K, SE, ΔK, age, sex, and the mean flat e or steep e. Statistical significance was set at *P* < 0.05.

## Results

### Demographics and corneal parameters

A total of 961 participants were included, with a mean age of 7.9 ± 2.9 years (56% male). The majority of participants (99.48%) exhibited with-the-rule anterior corneal astigmatism. The flat K and steep K were 42.28 ± 1.50 D and 45.31 ± 1.59 D, respectively, and ΔK was 3.03 ± 0.79 D. The SE of all participants was − 0.91 ± 3.00 D. The mean flat e and steep e (a chord length of 9.35 mm), were 0.73 ± 0.10 and 0.51 ± 0.21, respectively. Table 1 provides an overview of the participants’ demographic characteristics and corneal parameters.

**Table 1:** Summary of basic information of the participants

Parameter	Value
Age (years)	7.9 ± 2.9
Sex (%) Male Female	535 (56%) 426 (44%)
Corneal Astigmatism axis (%)	
With-the-rule (WTR)	956 (99.48%)
Against-the-rule (ATR)	2 (0.21%)
Oblique	3 (0.31%)
Flat K (D)	42.28 ± 1.50
Steep K (D)	45.31 ± 1.59
Flat e	0.73 ± 0.10
Steep e	0.51 ± 0.21
ΔK (D)	3.03 ± 0.79
SE (D)	−0.91 ± 3.00

### Distribution of corneal e values along flat and steep meridians

**Fig. 1:**
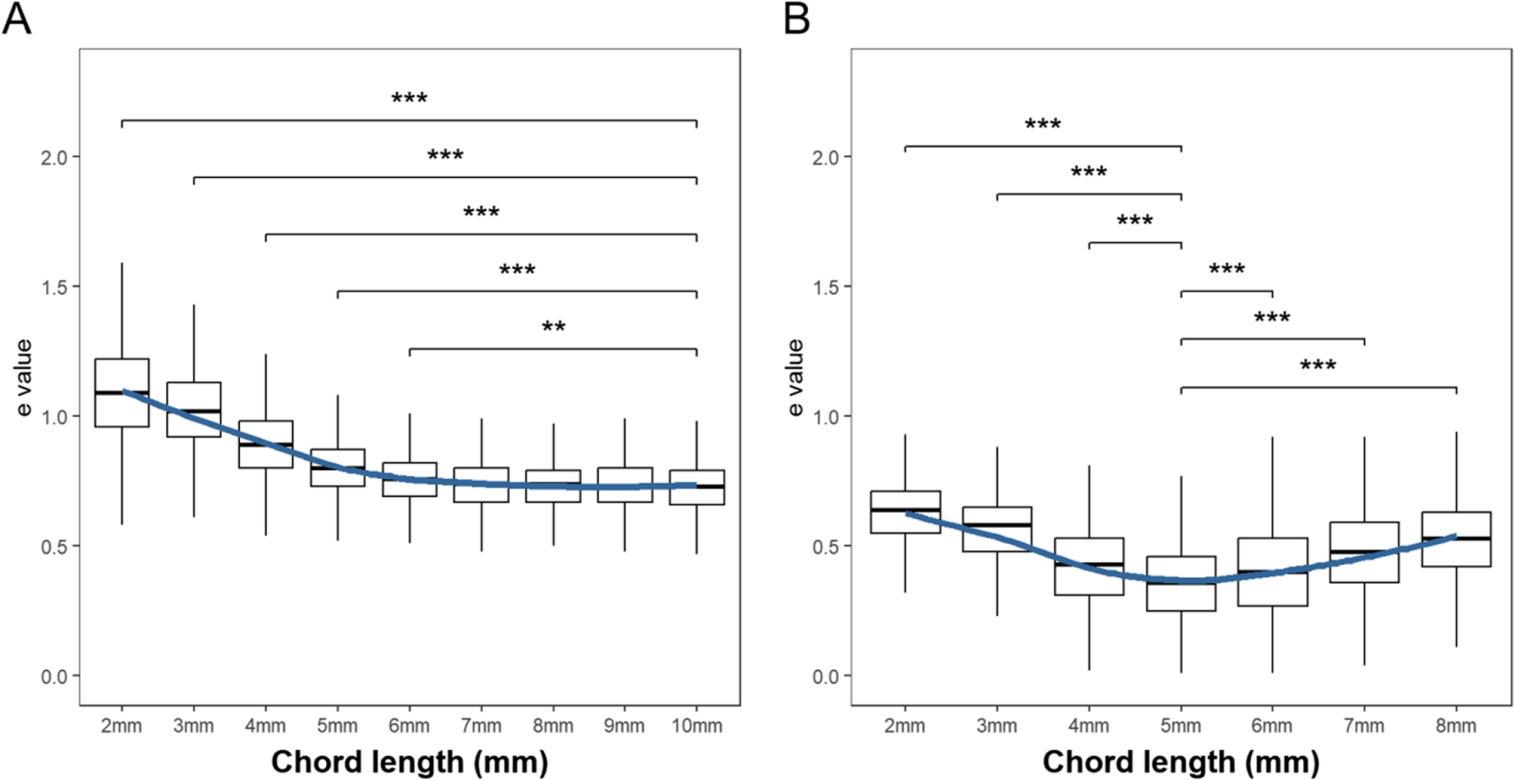
Distribution of corneal e values along the (**A**) flat and (**B**) steep meridians at various chord lengths. Asterisks indicate statistical significance based on post hoc comparisons: ***P < 0.001, **P < 0.01. Comparisons were made against the e value for a 10 mm chord length for the flat meridian (**A**) and a 5 mm chord length for the steep meridian (**B**)

As shown in Fig. 1, the distribution of e values along the flat and steep meridians displayed marked and asymmetric variations with increasing chord length. Notably, the e value at a chord length of 1 mm along both principal meridians deviated markedly from those at larger chord lengths. This may result from the steep central corneal curvature in high astigmatism and the inherent Limitations of conic fitting over very small chord lengths, where the corneal shape may locally diverge from an ideal conic profile. For completeness, 1 mm data are shown in Table 1, while Fig. 1 focuses on chord lengths from 2 mm. For the flat meridian, the sample sizes were 961 for 1–9 mm chord lengths and 581 for 10 mm. For the steep meridian, the sample sizes were 961 eyes for 1–6 mm, 797 eyes for 7 mm, and 296 eyes for 8 mm. No valid measurements were obtained for chord lengths of 9 mm and 10 mm for the steep meridian, likely due to eyelid coverage along the vertical meridian in with-the-rule astigmatism [28]. Data beyond a 9 mm chord length were excluded from the statistical analysis. Table 2 summarizes the distribution of corneal e values along the flat and steep meridians for different chord lengths.

In order to more precisely examine how the e value varies with chord length along the two principal meridians, we performed separate ANOVA’s for each meridian with post hoc comparisons using Tukey’s Honest Significant Difference (HSD) test (Table 2). For the flat meridian, e values varied significantly with chord length (F = 816.9, *P* < 0.001). Post-hoc Tukey tests indicated that e values at 10 mm differed from those at 2–6 mm (all *P* < 0.01), but not at 7–9 mm (all *P* > 0.05), exhibiting a smooth curvilinear pattern, which demonstrates an initial decrease followed by stabilization as the chord length increases. For the steep meridian, significant variation was observed at the 2–5 mm (F = 545.5, *P* < 0.001) and 5–8 mm (F = 104.2, *P* < 0.001) chord lengths, respectively. Post-hoc Tukey tests indicated that e values at a 5 mm chord length differed significantly from those at 2–4 mm (all *P* < 0.001) and 6–8 mm (all *P* < 0.001), suggesting a U-shaped pattern with an initial decrease in e values followed by an increase.

**Table 2:** Distribution of corneal e values along flat and steep meridians at different chord lengths

Meridian	Chord Length (mm)	N	Mean ± SD(95%CI)	Percentile						Tukey HSD	*P*
				P5	P25	P50	P75	P95	P99		
Flat	1	961	0.80 ± 0.08 (0.79–0.80)	0.68	0.77	0.81	0.84	0.88	0.90	-	-
	2	961	1.08 ± 0.25 (1.07–1.10)	0.68	0.96	1.09	1.22	1.45	1.66	−0.36	< 0.001
	3	961	1.02 ± 0.20 (1.01–1.04)	0.73	0.92	1.02	1.13	1.33	1.49	−0.30	< 0.001
	4	961	0.89 ± 0.15 (0.88–0.90)	0.66	0.80	0.89	0.98	1.13	1.27	−0.16	< 0.001
	5	961	0.80 ± 0.12 (0.80–0.81)	0.62	0.73	0.80	0.87	1.01	1.11	−0.08	< 0.001
	6	961	0.76 ± 0.11 (0.75–0.76)	0.60	0.69	0.76	0.82	0.95	1.03	−0.03	0.002
	7	961	0.74 ± 0.10 (0.74–0.75)	0.59	0.67	0.74	0.80	0.91	1.00	−0.01	0.600
	8	961	0.74 ± 0.09 (0.73–0.74)	0.59	0.67	0.74	0.79	0.90	1.00	−0.01	0.865
	9	961	0.73 ± 0.10 (0.73–0.74)	0.58	0.67	0.73	0.80	0.90	0.98	−0.01	0.998
	10	581	0.73 ± 0.10 (0.72–0.73)	0.57	0.66	0.73	0.79	0.89	0.97	Reference	Reference
Steep	1	961	1.48 ± 0.32 (1.46–1.50)	0.93	1.32	1.49	1.66	1.95	2.29	-	-
	2	961	0.62 ± 0.16 (0.61–0.63)	0.33	0.55	0.64	0.71	0.80	0.92	−0.26	< 0.001
	3	961	0.55 ± 0.15 (0.54–0.56)	0.27	0.48	0.58	0.65	0.74	0.78	−0.19	< 0.001
	4	961	0.42 ± 0.16 (0.41–0.43)	0.14	0.31	0.43	0.53	0.64	0.75	−0.05	< 0.001
	5	961	0.36 ± 0.16 (0.35–0.37)	0.10	0.25	0.36	0.46	0.64	0.78	Reference	Reference
	6	961	0.40 ± 0.18 (0.39–0.41)	0.12	0.27	0.40	0.53	0.70	0.82	0.04	< 0.001
	7	797	0.47 ± 0.17 (0.46–0.48)	0.19	0.36	0.48	0.59	0.73	0.84	0.11	< 0.001
	8	296	0.52 ± 0.16 (0.51–0.54)	0.26	0.42	0.53	0.63	0.76	0.85	0.16	< 0.001

Note: P values are from Tukey Honest Significant Difference (HSD) post hoc tests following one-way ANOVA performed independently for each meridian. Comparisons were made against the e value at 10 mm for the flat meridian and 5 mm for the steep meridian.

### Factors associated with mean flat e or steep e values

Table 3 summarizes the results of the simple and multiple linear regression analysis examining the relationship between mean flat e or steep e and studied variables. Simple linear regression revealed that the mean flat e was correlated with the flat K and ΔK, and these associations were also significant in the multiple linear regression model. For the mean steep e, simple linear regression indicated associations with sex (male = 1, female = 0), steep K, and ΔK, but only sex remained significant in the multiple linear regression model.

**Table 3:** Simple and multiple linear regression analysis of mean flat e or steep e

Dependent variables	Independent variables	Simple linear regression		Multiple linear regression	
		Coefficient (95% CI)	P	Coefficient (95% CI)	P
Mean flat e	Age	−0.001 (− 0.003, 0.002)	0.582	—	—
	Sex	−0.011 (− 0.023, 0.002)	0.097	—	—
	Flat K	−0.016 (− 0.020, − 0.011)	< 0.001	−0.013 (− 0.017, − 0.009)	< 0.001
	ΔK	0.040 (0.033, 0.048)	< 0.001	0.037 (0.030, 0.045)	< 0.001
	SE	−0.002 (− 0.004, 0.001)	0.143	—	—
Mean steep e	Age	−0.003 (− 0.007, 0.002)	0.265	—	—
	Sex	−0.046 (− 0.072, − 0.019)	< 0.001	−0.043 (− 0.070, − 0.017)	0.001
	Steep K	0.000 (0.000, 0.016)	0.047	0.002 (− 0.007, 0.011)	0.597
	ΔK	0.019 (0.002, 0.035)	0.026	0.016 (− 0.002, 0.034)	0.074
	SE	0.003 (− 0.002, 0.007)	0.231	—	—

Note: Sex was coded as male = 1 and female = 0

## Discussion

Comprehensive data on anterior corneal e values along both principal meridians across various chord lengths in pediatric and adolescent Chinese populations with moderate to high corneal astigmatism are limited. Our study provides a large-scale, detailed characterization of meridional e value patterns in this population, addressing an important gap in pediatric corneal morphology research. The sample size of 961 participants in our study was greater than previous studies examining corneal e values in this population [3, 5]. Specifically, Chui et al. [3] included 22 children aged 11.2 ± 2.2 years, and Li et al. [5] included 143 children aged 8–19 years with a mean astigmatism of − 0.78 ± 0.52 D. In the current study, the majority (99.48%) of participants exhibited with-the-rule astigmatism, which is consistent with the common occurrence of with-the-rule astigmatism in children and adolescents [29]. In our study, the flat K was 42.28 ± 1.50 D, and the steep K was 45.31 ± 1.59 D. Compared with previous studies [8, 17] the higher steep K in our cohort likely reflects the inclusion of participants with moderate-to-high astigmatism. This highlights that in patients with high corneal astigmatism, the increase in ΔK is primarily driven by steepening of the steep corneal meridian rather than flattening of the flatter meridian.

In our study, the mean flat and steep e values were 0.73 ± 0.10 and 0.51 ± 0.21, respectively. These values differ from prior studies primarily due to differences in the magnitude of astigmatism, sample size, and participant characteristics. For example, Li et al. [5] excluded participants with astigmatism greater than 1.50 D and reported mean flat and steep e values of 0.65 ± 0.08 and 0.45 ± 0.16, respectively. Differences in instrumentation, ethnicity, and age may also play a role. For example, Gruhl et al. [6] examined 106 German individuals aged 9 to 52 years, excluding participants with astigmatism greater than 1.50 D, and reported a mean flat e value of 0.54 ± 0.11 and a mean steep e value of 0.52 ± 0.13 using the Keratograph 5 M. These variations highlight the potential influence of study design, population characteristics, and measurement devices on corneal e values. A detailed summary of previous study designs is provided in Table S2 to contextualize these comparisons.

Notably, in our study, the e values at 1 mm along both principal meridians exceeded 1. This is because the Medmont E300 derives the measured e values by fitting the anterior corneal surface to theoretical surface shapes through a conic fitting approach (ellipse, parabola, or hyperbola, as described in the user manual). Although corneal e values generally range from 0 to 1, values greater than 1 may appear at very small chord lengths (~ 1 mm), reflecting a locally hyperbolic corneal profile.

Most previous studies have used a single e value to represent the overall corneal shape, typically obtained directly from the instrument software without any additional information. However, this study revealed that corneal e value varies substantially with chord length. Similar to the e value, the Q value measures the rate of change in corneal curvature [2] with Q = − e^2^. Previous research has demonstrated that the corneal Q value changes with chord length [1, 30–33]. For instance, Zhang et al. [33] reported Q values of − 0.09 ± 0.21, − 0.14 ± 0.16, − 0.15 ± 0.13, − 0.17 ± 0.11, and − 0.20 ± 0.11 at chord lengths of 3, 4, 5, 6, and 7 mm, respectively. This indicates a negative correlation between Q value and chord length, which corresponds to a positive relationship between e value and chord length.

In the corneal periphery, corneal astigmatism may either stabilize, increase, or decrease [29]. Our data revealed distinct meridian-specific patterns: the flat corneal meridian exhibited a smooth curvilinear pattern, characterized by a gradual reduction in e values from the central to the peripheral regions, eventually stabilizing, whereas the steep meridian showed a characteristic U-shaped progression.

No correlation was observed between age and the mean e values of both meridians. Previous cross-sectional studies [4, 15, 18, 34] have reported that e values decreased with age, while longitudinal studies [26, 35] have reported the opposite trend. Additionally, we observed sex-related differences, with females exhibiting higher e values than males, consistent with previous research [18, 26]. Further analysis revealed that the flat K (t = − 6.23, *P* < 0.001), steep K (t = − 6.39, *P* < 0.001), and ΔK (t = − 0.78, *P* < 0.001) varied significantly, indicating potential sex-related differences in corneal morphology. However, as with prior studies, the biological rationale for this association is not yet well understood and requires further exploration.

No significant correlations were found between the mean flat e or steep e and SE, which is consistent with some studies [1, 19, 36], but conflicting with others [4, 15, 35, 37]. This discrepancy may result from differences in population characteristics (e.g., the inclusion of participants with moderate to high astigmatism in our study), age, or measurement methods, warranting further investigation. Furthermore, we identified a significant negative correlation between mean flat e and flat K, but no correlation was found between mean steep e and steep K. Previous studies have suggested that a steeper cornea is associated with a greater rate of curvature change from the center to the periphery [15, 36] although others [34] found no such correlation.

A positive correlation was observed between mean flat e and ΔK, with no significant correlation for mean steep e. These results align with González-Méijome et al. [30], who also measured corneal Q values along the two principal meridians. Other studies [1, 15] have similarly reported associations between corneal shape factors and astigmatism, highlighting the complex interplay between corneal morphology and toricity.

While the observed changes in e values were small but statistically significant, their isolated clinical impact may be limited. Nevertheless, subtle variations in corneal e values might contribute cumulatively to corneal shape characterization and refractive outcomes, warranting further clinical investigation.

This study has several limitations. First, corneal data were available only up to 8 mm along the vertical meridian, the steeper meridian in most participants, due to eyelid obstruction. Second, the focus on participants with with-the-rule astigmatism may limit the generalizability of our findings to other types of astigmatism. Third, recruitment from a single center could introduce selection bias and restrict broader applicability. Future studies should develop improved tools for peripheral corneal measurements and include more diverse populations to enhance the generalizability of results.

## Conclusion

In summary, this study explored the distribution and related factors of anterior corneal e value in Chinese children and adolescents with moderate to high corneal astigmatism. The findings provide valuable insights for contact lenses design and fitting. For corneal rigid lenses, orthokeratology, and scleral lenses, optimal alignment with the anterior corneal surface may be improved not only by incorporating back surface toricity but also by considering meridionally varying e values, especially in eyes with significant astigmatism. This strategy may enhance lens centration, stability, and comfort. In soft lens designs, adjusting posterior surface asphericity based on meridional corneal profiles may also improve fit and visual performance. These clinical implications should be considered in future lens development and may also inform diagnosis and surgical planning in this population, especially given the unique corneal characteristics of Chinese pediatric patients.

## Supplementary Information

Supplementary Material 1.

## Data Availability

The datasets are not publicly available due to patient privacy concerns but can be provided by the corresponding author upon reasonable request and approval from the institutional ethics committee.
