# Supplementary material for: Corneal eccentricity and related factors in Chinese children and adolescents with astigmatism
Source: BMC Ophthalmol. 2025 Oct 7;25:550. doi: 10.1186/s12886-025-04402-3 (PMC12502173; doi:10.1186/s12886-025-04402-3)
Supplement: Supplementary file 1 — Supplementary Material 1. [file 12886_2025_4402_MOESM1_ESM.docx]

| Table S1. Age group distribution of participants | | |
| --- | --- | --- |
| Age | N | Percentage (%) |
| 3-5 | 126 | 13.1 |
| 6-8 | 439 | 45.7 |
| 9-11 | 265 | 27.6 |
| 12-14 | 91 | 9.5 |
| 15-18 | 40 | 4.2 |

Table S2. Summary of previous studies on corneal shape and e values

| Author (Year) | Sample size | Age (years) | Population/Place | Device | Reported e value | Chord diameter |
| --- | --- | --- | --- | --- | --- | --- |
| Chui et al. (2005)^1^ | 22 | 11.2±2.2 | China | Medmont E300 (Medmont, Australia) | 0.66±0.11 | 9.0 mm |
| Patel et al. (2009)^2^ | 62 | 21–30, 25±2.7 | New Zealand | Orbscan II (Bausch & Lomb, Salt Lake City, USA) | Anterior: 0.52  Posterior: 0.50 | NA |
| Minami et al. (2012)^3^ | 70 | 37–70, 69.4±9.8 | Japan | TMS-5 (Tomey, Japan) | 0.24±0.25 | 6.0 mm |
| Asgari et al. (2013)^4^ | 4266 | 40–64, 50.7±12.02 | Iran | Pentacam HR (Oculus, Germany) | 0.27±0.63 (Range: −1.42–1.32) | NA |
| Piñero et al. (2017)^5^ | 107 | 23–65, 47.8 | Spain | VX120 system (Visionix-Luneau Technologies, France) | 0.34±0.31 (Range: −1.16–0.83) | 3.0 mm |
| Heydarian et al. (2018)^6^ | 2533 | 6–90, 36.23±18.46 | Iran | Pentacam HR (Oculus, Germany) | 0.53 (95% CI: 0.50–0.54) | NA |
| Yoshida et al. (2022)^7^ | 1215 | 40–90, 67.7±8.4 | Japan | Pentacam (Oculus, Germany) | 0.46±0.18 (Range: −0.10–0.84) | 6.0 mm |
| Li et al. (2022)^8^ | 143 | 8–19, 10.7±2.2 | China | Medmont E300 (Medmont, Australia) | Flat e: 0.65±0.08 (Range: 0.46–0.85)  Steep e: 0.45±0.16 (Range: 0.05–0.82) | NA |
| Gruhl et al. (2023)^9^ | 106 | 9–52, 22.16±8.96 | Germany | Keratograph 5M (Oculus, Germany) | Flat e: 0.54±0.11 (Range: 0.10–0.84)  Steep e: 0.52±0.13 (Range: −0.06–0.81) | NA |
| Barrio et al. (2024)^10^ | 113 | 20–63, 25.00±8.46 | Spain | Pentacam tomographer (Oculus, Germany) | 0.53±0.09 | NA |
| Present study | 961 | 3-18, 7.90±2.86 | China | Medmont E300 (Medmont, Australia) | Flat e: 0.73±0.10 (Range: 0.35–1.05)  Steep e: 0.51±0.21 (Range: 0.04–1.78) | 9.35 mm |

Abbreviations: NA = not available.


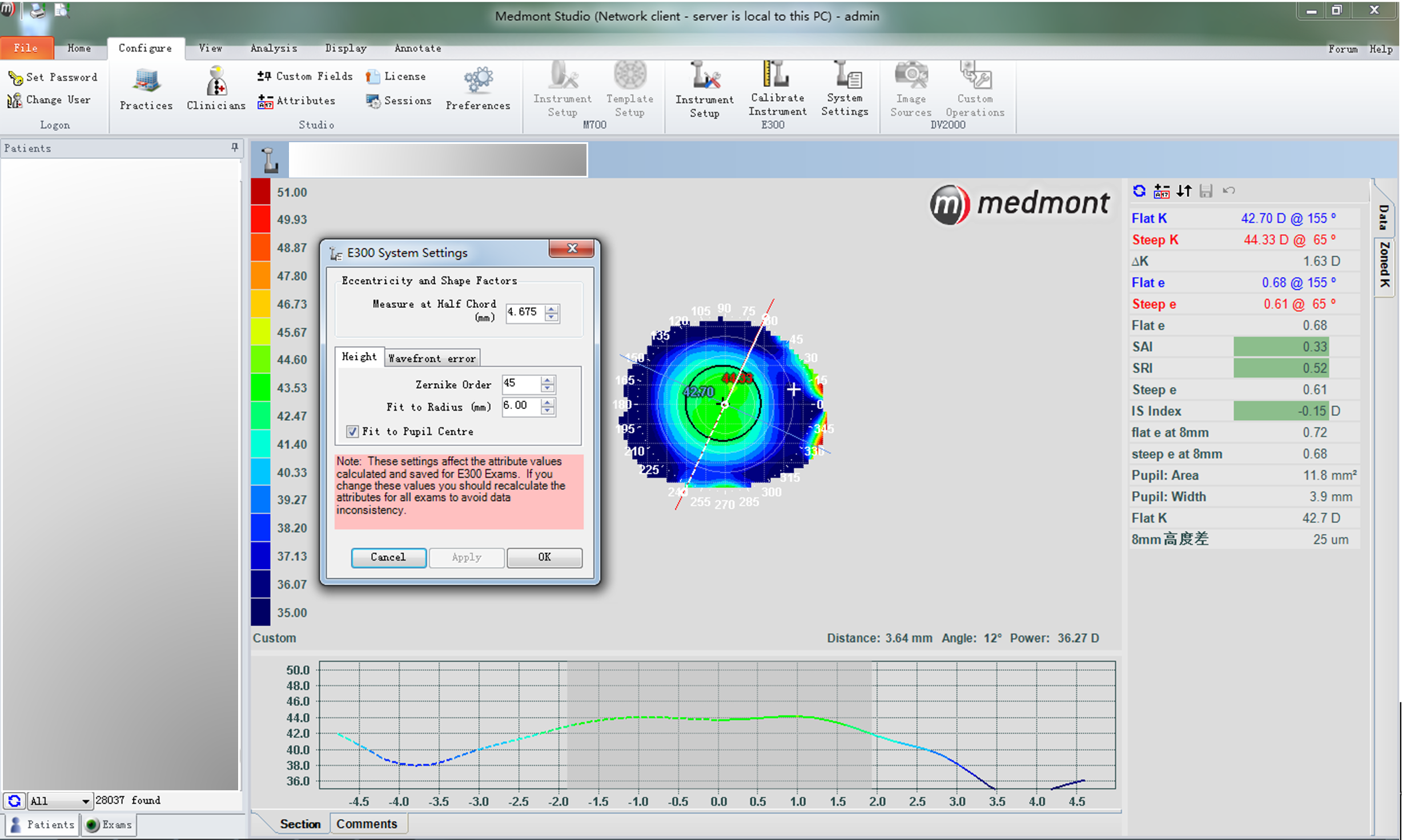


**Supplementary Figure 1.**

Representative screenshot from the Medmont E300 topographer interface.
The default eccentricity (e) value displayed by the software is based on a 9.35 mm chord length diameter (or 4.675 mm half chord length as presented), which can be verified in the system configuration panel. The same interface also shows key corneal parameters, including Flat K, Steep K, their respective axes, and ΔK, all summarized in the measurement panel on the right side.


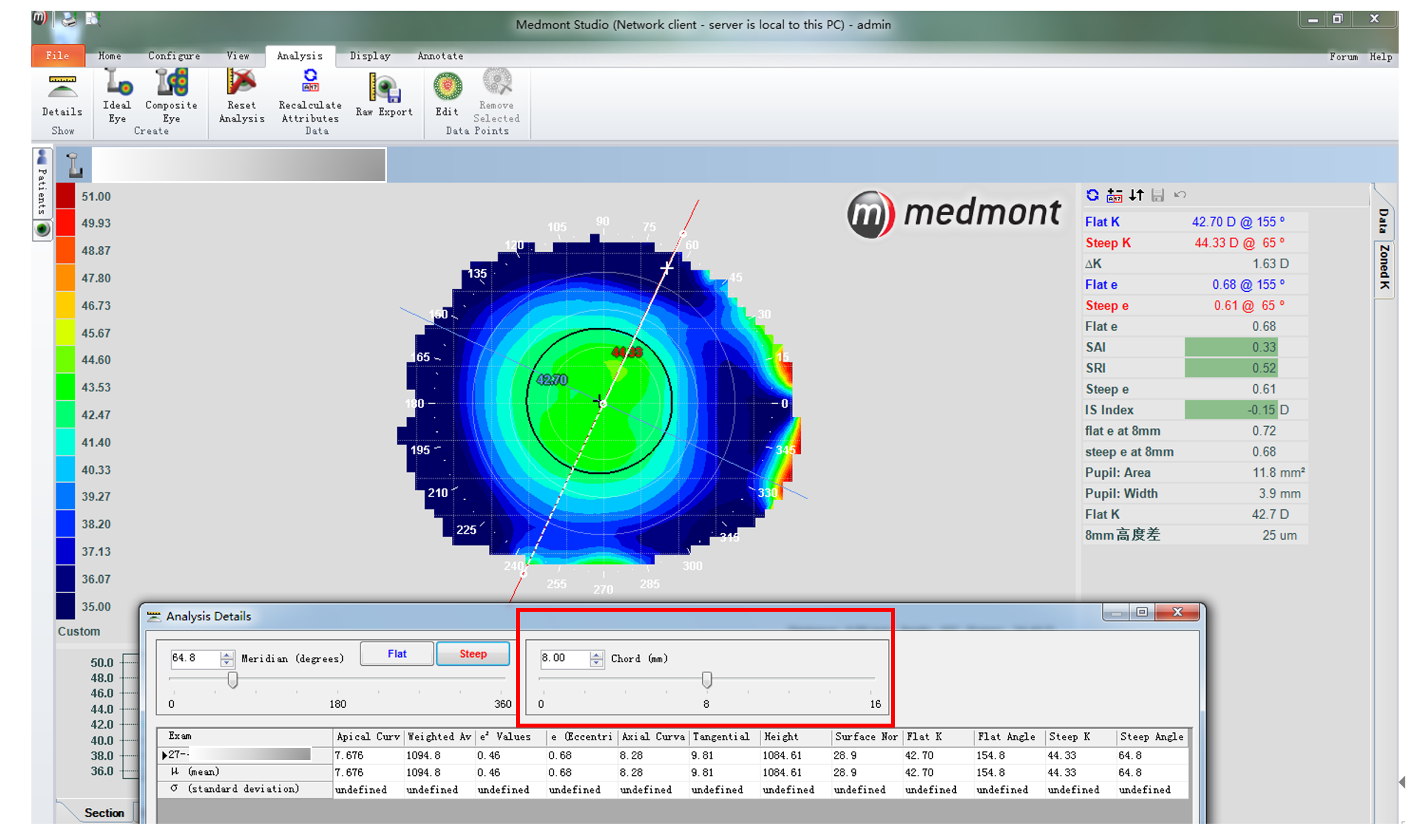


**Supplementary Figure 2.**

Eccentricity (e) values across various chord lengths (1–10 mm) can be manually obtained by adjusting the chord length in the analysis settings, as indicated by the red box.

**References:**

1. Chui WS, Cho P. A Comparative Study of the Performance of Different Corneal Topographers on Children with Respect to Orthokeratology Practice: *Optom Vis Sci*. 2005;82(5):420-427. doi:10.1097/01.OPX.0000162642.24885.71

2. Patel H, Patel D, McGhee C. Identifying relationships between tomography-derived corneal thickness, curvature, and diameter and in vivo confocal microscopic assessment of the endothelium in healthy corneas of young adults. *Eye Lond Engl*. 2009;23(2):270-278. doi:10.1038/sj.eye.6703091

3. Minami K, Kataoka Y, Matsunaga J, Ohtani S, Honbou M, Miyata K. Ray-tracing intraocular lens power calculation using anterior segment optical coherence tomography measurements. *J Cataract Refract Surg*. 2012;38(10):1758-1763. doi:10.1016/j.jcrs.2012.05.035

4. Asgari S, Hashemi H, Mehravaran S, et al. Corneal Refractive Power and Eccentricity in the 40- to 64-Year-Old Population of Shahroud, Iran. *Cornea*. 2013;32(1):25-29. doi:10.1097/ICO.0b013e31824d0e40

5. Piñero DP, López-Navarro A, Cabezos I, Fez DD, Caballero MT, Camps VJ. Corneal Topographic and Aberrometric Measurements Obtained with a Multidiagnostic Device in Healthy Eyes: Intrasession Repeatability. *J Ophthalmol*. 2017;2017:1-9. doi:10.1155/2017/2149145

6. Samira H, Hassan H, Fereshteh S, et al. The normal distribution of corneal eccentricity and its determinants in two rural areas of north and south of Iran. *J Curr Ophthalmol*. 2017;30(2). doi:10.1016/j.joco.2017.11.006

7. Yoshida Y, Ono K, Tano T, et al. Corneal Eccentricity in a Rural Japanese Population: The Locomotive Syndrome and Health Outcome in Aizu Cohort Study (LOHAS). *Ophthalmic Epidemiol*. 2022;29(5):531-536. doi:10.1080/09286586.2021.1968004

8. Li C, Zeng L, Zhou J, Wang B, Chen Z. To Achieve a Bullseye: Factors Related to Corneal Refractive Therapy Orthokeratology Lens Toricity. *J Clin Med*. 2022;11(19):5635. doi:10.3390/jcm11195635

9. Gruhl J, Widmer F, Nagl A, Bandlitz S. Factors influencing treatment zone size in orthokeratology. *Contact Lens Anterior Eye*. 2023;46(4):101848. doi:10.1016/j.clae.2023.101848

10. Barrio AR, Antona B, Morago-Villanueva S, Martínez-Arribas V, González-Pérez M. Intrasession repeatability and agreement of the anterior corneal assessment provided by a multidiagnostic device. *Clin Exp Optom*. Published online March 17, 2024:1-7. doi:10.1080/08164622.2024.2324978
